# The Musculoskeletal Anatomy of the Komodo Dragon’s Hindlimb (*Varanus komodoensis*, Varanidae)

**DOI:** 10.3390/ani15010035

**Published:** 2024-12-26

**Authors:** Anna Tomańska, Martyna Stawinoga, Tomasz Gębarowski, Maciej Janeczek, Joanna Klećkowska-Nawrot, Karolina Goździewska-Harłajczuk, Maciej Dobrzyński

**Affiliations:** 1 Department of Biostructure and Animal Physiology, Faculty of Veterinary Medicine, Wrocław University of Environmental and Life Sciences, Kożuchowska St. 1, 51-631 Wrocław, Poland; 2 Veterinary Biotechnology Student Science Club “Refectio”, Department of Biostructure and Animal Physiology, Faculty of Veterinary Medicine, Wrocław University of Environmental and Life Sciences, Kożuchowska St. 1, 51-631 Wrocław, Poland; 3 Department of Pediatric Dentistry and Preclinical Dentistry, Wroclaw Medical University, Krakowska 26, 50-425 Wrocław, Poland

**Keywords:** anatomy, lizards, Komodo dragon, orthopedics

## Abstract

The Komodo dragon (*Varanus komodoensis*) is the largest extant lizard and is classified as an endangered species. Despite its rarity, anatomical studies on this species remain limited, hindering a comprehensive understanding of its biology and evolutionary traits. This research presents a detailed anatomical and histological examination of the pelvic limb of a female Komodo dragon, providing valuable insights into the musculoskeletal system of this species. A series of measurements and observations were made on the bones of the pelvic limb, including the femur, tibia, fibula, and pes, all of which are essential for supporting the animal’s large body size and facilitating its predatory behavior. This study also highlights the diverse muscle architecture, where large muscle masses are associated with the femoral retractors and ankle plantarflexors. Histological analysis of the muscle fibers revealed significant variability in fiber diameters, supporting the functional adaptation of the Komodo dragon’s limbs for high-speed ambush predation. This research provides important morphological data that could inform clinical practices, including orthopedic procedures and physiotherapy for Komodo dragons in zoological settings. Additionally, these findings shed light on the evolutionary patterns inherited from the species’ ancestors, which contributed to the development of its distinctive biological adaptations.

## Simple Summary

This study provides a detailed examination of the musculoskeletal anatomy of the Komodo dragon’s hindlimbs, with a focus on structural features related to posture and locomotion. Anatomical dissection was primarily performed to investigate all regions of the pelvic limb. Radiographic imaging was employed complementarily to analyze the overall skeletal structure, while histological analysis was applied to examine the muscle tissue and fiber architecture. The muscles demonstrated a dense fiber arrangement, leading to a compact and firm structure with minimal adipose tissue and a well-developed connective tissue sheath. The muscle fiber diameters ranged from 11 to 220 µm, underscoring significant diversity in fiber architecture. These findings improve our understanding of the Komodo dragon’s pelvic limb anatomy and open new avenues for research into its functional adaptations, with broader implications for its biological research. These results contribute valuable anatomical insights, emphasizing the pivotal role of biomechanics in the evolutionary adaptations of Komodo dragons, particularly in sustaining a sprawling limb posture. This research is especially relevant for clinicians, including orthopedic surgeons, who are involved in limb-related procedures for this species.

## 1. Introduction

The Komodo dragon (*Varanus komodoensis* [Ouwens, 1912]) [1] is the largest extant lizard, currently classified as endangered due to habitat loss and human activity. As an island-endemic species, its survival is particularly vulnerable to environmental changes [2] and human encroachment [3]. Despite its conservation status, a significant lack of anatomical data hampers a thorough understanding of its biology. The limited representation of the species in zoos further complicates this issue. Consequently, accurate data collection is essential to unravel the evolutionary morphology of these reptiles, especially with the advancement of technologies within conservation frameworks [4]. Many aspects of their biology remain inadequately understood, such as the maximum size and habitat preferences [5]. The Komodo dragon is a gigantic species, and its ancestors show minimal morphological variation, which contributes to comparative studies in evolutionary biology [6]. The minimal morphological variation observed in the ancestors of *V. komodoensis* positions the Komodo dragon as a “living fossil”, offering significant insights into the evolutionary transition from a quadrupedal to an upright posture in vertebrates. While research has primarily focused on the forelimb, the hindlimb requires a more focused and distinct analysis [7,8]. It is essential to note that this study is based on a single individual, highlighting the need for further comprehensive morphological data on *V. komodoensis* to deepen our understanding.

Most lizards and crocodiles exhibit a sprawling limb posture, with bone loading increasing as the femoral bone axis rotates. This stress amplifies with body size, suggesting that the limb bone width in these animals will scale with a greater allometric relationship compared to other species [4]. The limb structure is primarily involved in generating propulsive force, which is essential for running economy, maximum speed, acceleration, gait specificity, and endurance [9]. For land-dwelling animals, especially large organisms, the biomechanical architecture of pelvic limbs presents a significant challenge [10]. Allometric physical values in large-bodied animals offer valuable insights into evolutionary patterns, particularly regarding the adaptations of lizards to a sprawling limb posture.

Ecomorphological studies reveal distinct trends in the adaptation of monitor lizards to their environments. While it is generally observed that species in open habitats tend to be faster, monitor lizards exhibit a stronger correlation between their morphology and functional adaptations, differentiating them from other lizard species [11]. The functionality of their anatomy is further evident in the variability in muscle fiber orientation and length. These factors influence the range, speed, and force of sarcomere shortening, which is critical for performing specific limb movements [12].

In monitor lizards, limb length typically scales with body size, with larger individuals having proportionally thicker limbs. However, the growth of the feet occurs at a different rate compared to the pelvic limbs [13]. These morphological differences have functional and behavioral consequences, which are crucial for paleoichnological taxonomy. They provide insights into the posture and locomotion of extinct species, such as Triassic pseudosuchians [8].

The adult Komodo dragon hunts prey significantly larger than itself from an ambush position. Therefore, achieving high speed over a relatively short distance is critical for hunting success. The pelvic limbs play a key role as the “engine” that initiates body movement [14]. As a quadruped [15], the architectural and biomechanical features of the muscular system of the Komodo dragon have been elucidated through comparative methods, particularly in relation to *Alligator mississippiensis* [16] and other quadrupedal animals, including mammals, amphibians, and birds [17].

In comparison to the Komodo dragon, a general principle emerges, suggesting that the greatest muscle mass relative to body size is found in the hindlimb retractors and ankle flexors, followed by the knee extensors, which are slightly more massive than the knee flexors, and then the ankle extensors. The smallest muscle mass is associated with the femur adductors, femur protractors, and femur abductors [10]. These findings align with evidence of positive allometry in the muscle mass or physiological cross-sectional area of the femur adductors, knee flexors, and ankle plantarflexors [17].

The morphological data on the pelvic limb anatomy of the Komodo dragon presented here can also support clinical tasks, particularly for orthopedic surgeons who have described procedures for limb surgeries in this species [18]. Confirming the previously observed disproportion in muscle mass distribution and functionality, these insights may inform the development of physiotherapeutic techniques for animals in zoological settings. Techniques such as the Wolfe Kinetic Technique™ (Arvada, United States) have been successfully implemented, showing positive outcomes in improving gait and limb functionality [7].

This is particularly significant, as there are indications suggesting that the additional muscle mass in Komodo dragons enhances their relative force-generating capacity, which aids in supporting the substantial body weight on all their limbs [17].

## 2. Materials and Methods

The Department of Biostructure and Animal Physiology at the University of Life Sciences in Wrocław is conducting fundamental research aimed at providing valuable morphological data on the biology of various species. As part of this study, a female Komodo dragon (*Varanus komodoensis*) specimen, obtained from the Wrocław Zoological Garden (Wrocław, Poland) following natural death, is being examined through anatomical dissection and histological analysis. The specimen was first injected with formalin and subsequently fully immersed in a formalin solution for preservation. The female Komodo dragon was 7 years old, weighed 30 kg, and had a snout-to-vent length (SVL) of 93.6 cm, a head length (HL) of 18.0 cm, and an ear opening-to-snout distance (EOS) of 15.4 cm. This individual was housed at the Wrocław Zoological Garden (Wrocław, Poland). Post mortem, the body was preserved by formalin injection and further secured by immersion in a formalin tank. Following this preservation process, radiographic imaging was performed using X-rays, with images analyzed using the CR 7 VET scanner (iM3, Lane Cove, NSW, Australia) and Vet-Examplus 9.3.0 software (iM3 Pty Ltd., Lane Cove, NSW, Australia).

Subsequently, both pelvic limbs were severed from the pelvis at the hip joints. The muscles were carefully dissected and described along the entire length of each limb. The caudofemoral long muscle (*m. caudofemoralis longus*) was not detailed in this study due to the different methodological approach required and warrants further analysis. Dissection began by exposing the muscles beneath a thick fascial layer using a scalpel and forceps. Two histological preparations were made from the skin, fixed in 50 mL vials containing a 4% formalin solution. The tissue was dehydrated through a series of alcohol solutions and embedded in paraffin. The tissue sections were cut both transversely and longitudinally, producing slices of 5–7 µm thickness using a microtome. These sections were stained with hematoxylin and eosin (HE).

The muscles were sequentially isolated and described. Detailed zoometric measurements were taken, including the muscle mass, length from origin to insertion, and circumference at the widest point of key limb muscles.

Measurements were conducted using a flexible tape measure, with two independent readings validated against dimensions obtained using a standardized archaeological ruler made of synthetic paper. The values were then averaged using a weighted mean and rounded to one decimal place.

The distribution of muscle fiber diameters, circumference, length, and weight across different muscles in the pelvic limbs was visualized with Datawrapper (https://www.datawrapper.de, accessed on 12 October 2024). Illustrations of the muscles were produced using Adobe Illustrator (Adobe Inc., 2019, San Jose, CA, USA).

From the isolated muscles, histological samples were collected, with three fragments measuring 1 cm × 1 cm × 1 cm taken from various regions of each muscle. Histological preparations were fixed in 50 mL vials containing a 4% formalin solution. Muscle tissue was subsequently dehydrated in a series of alcohol solutions and embedded in paraffin. The tissue sections were cut both transversely and longitudinally to the muscle fibers, yielding slices of 7–10 µm thickness using a microtome. These sections were stained with hematoxylin and eosin (HE) to enable morphometric analysis, performed using a Nikon Eclipse 80i microscope (© 2023 Nikon Instruments Inc., Melville, NY, USA) and NIS-Elements AR software (Ver. 4.50, © Nikon Instruments Inc., Melville, NY, USA).

The arrangement of bones was also based on comparisons with the skeleton of another *V. komodoensis* specimen from the collection of the Nature Museum at the Faculty of Biology and Animal Science, Wrocław University of Environmental and Life Sciences (Wrocław, Poland).

## 3. Results

The proximal-to-distal length of the hindlimb, from the hip joint to the claw tip, was 37.9 cm, with the pes (measured from the tarsocrural joint to the distal digits) accounting for 12.7 cm. The pes made up 33.5% of the total hindlimb length, while the proximal segments (hip, thigh, and shank) comprised 66.5%. The hindlimbs were approximately 6 cm longer than the forelimbs, resulting in a pelvic limb-to-forelimb ratio of 1.32. The pes-to-hand ratio was 1.84, indicating that the pes is nearly twice as long as the manus. The functional limb index, calculated as the ratio of the hindlimb length to snout-to-vent length (SLV), was 40.5, reflecting the hindlimb’s strong contribution for force generation. The stride potential, defined as twice the hindlimb length, was 75.8 cm, indicating the potential stride length.

**Figure 1:**
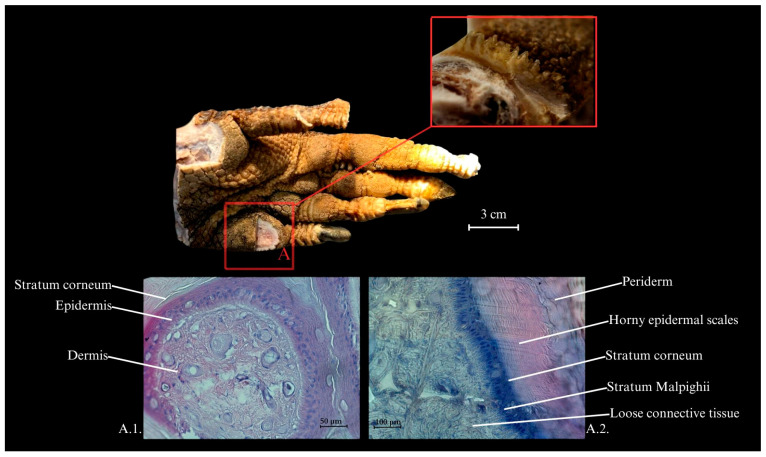
Skin of the sole of the Komodo dragon’s pes. (**A**). Cross-sectional view of the skin emphasizing the horny epidermal scales (squamae epidermales cornificatae). The histological image was stained with hematoxylin and eosin (HE). (**A.1**)—a cross-section of the skin with annotated stratum corneum, epidermis, and dermis (corium), Mag 100×. (**A.2**)—a longitudinal section of the skin with annotations for periderm (peridermis), horny epidermal scales (squamae epidermales cornificatae), stratum corneum, stratum Malpighii, and loose connective tissue (textus connectivus laxus), Mag 200×.

The limb is covered in scales, each containing small dermal ossicles (osteoderms) beneath them [19,20], which range in color from beige to dark brown. These scales form characteristic keratinized ridges. The horny layer (*stratum corneum*) is highly developed, while the dermis is rich in collagen fibers. On the sole of the pes, keratinized pads are present alongside smaller scales. The amount of adipose tissue is minimal, and beneath the skin lies a substantial layer of connective tissue, particularly prominent in the metatarsal region (*pars metatarsalis*) (Figure 1).

**Figure 2:**
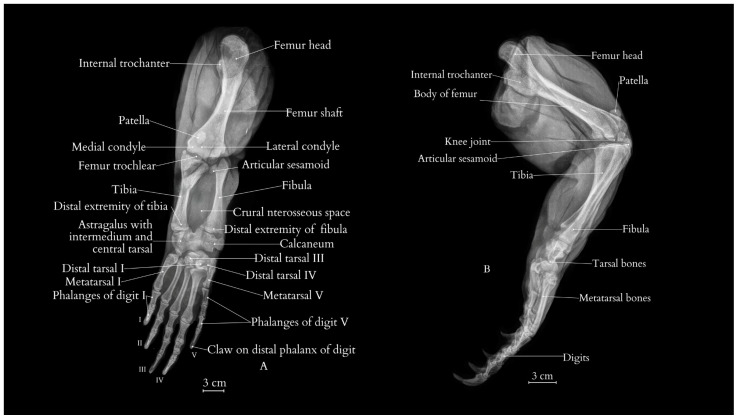
The posterio-anterior (**A**) and lateral (**B**) view of the right pelvic limb of the Komodo dragon (X-rays). The following anatomical structures are visible: femur with its internal trochanter and shaft; condyles and intercondylar groove; tibia with its distal extremity; calcaneum; fibula with the distal epiphysis of the fibula; astragalus with intermedium and central tarsal bone; distal tarsal III; metatarsal V; metatarsal I; I—digit I; II—digit II; III—digit III; IV—digit IV; and V—digit V.

**Figure 3:**
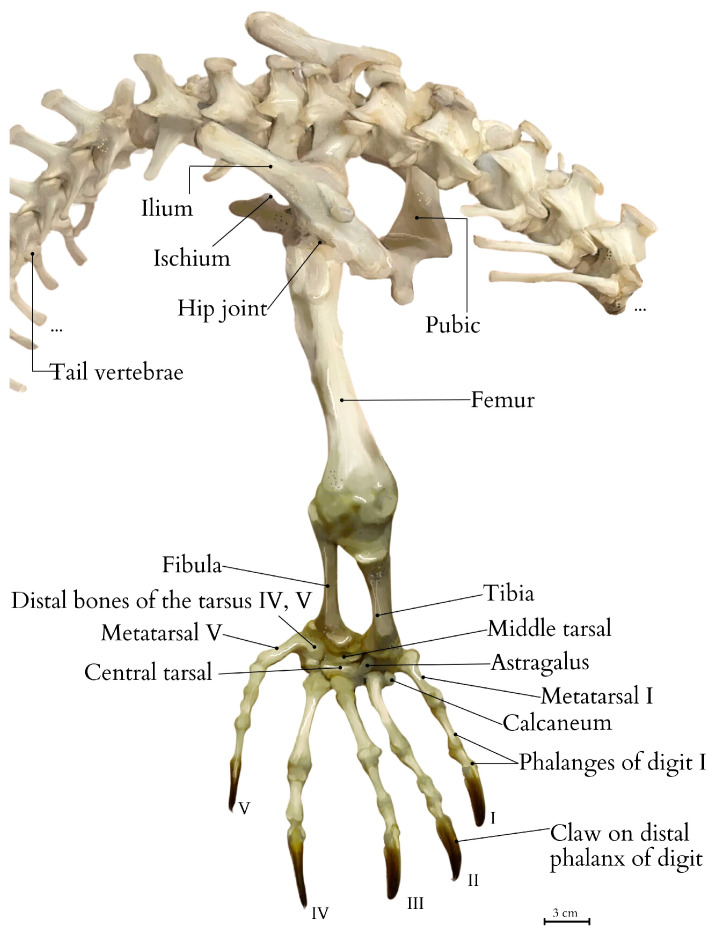
The skeletal structure of the right pelvic limb of the Komodo dragon, emphasizing its connection to the pelvis. It includes the pelvis, femur, tibia, fibula, metatarsals, calcaneum bone, and digits. Labels: I—digit I; II—digit II; III—digit III; IV—digit IV; and V—digit V. The illustration is based on the anatomical examination and photographic documentation of the comparative specimen from the collections of the Nature Museum at the Faculty of Biology and Animal Science, Wrocław University of Environmental and Life Sciences (Wrocław, Poland) [21]. Created by A. Tomańska.

The bones of the pelvic limb include the femur, tibia, fibula, tarsal bones, metatarsals, and phalanges. The bones of the hindlimb are notably sturdy and broad, providing significant support for the animal’s mass. The knee joint includes two sesamoid bones enhancing articulation. The lizard’s foot is characterized by a well-developed pes with five digits, each ending in thick, robust claws (Figure 2 and Figure 3).

The femur’s shape is wide and well defined, with a pronounced internal trochanter at its proximal end, indicating strong muscle attachment sites. It also displays prominent condyles, which are rounded and suggest a robust joint structure. A substantial interosseous space between the tibia and fibula facilitates effective torsional stress distribution, helping balance the forces generated by muscles attached to the femur.

Our findings are consistent with the anatomical descriptions provided by Dick and Clemente, confirming the detailed structure of the femoral musculature [16]. The musculature of the limb is dense and compact, with thick nerves innervating it, forming bundles of fibers that radiate outward.

**Figure 4:**
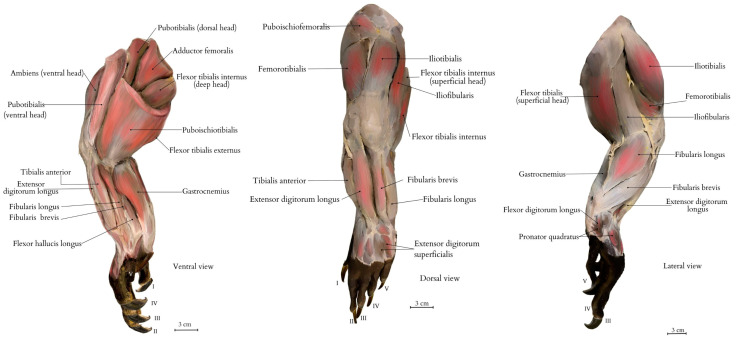
Muscular anatomy of the right pelvic limb, including the following muscles: pubotibial muscle (*m. pubotibialis*), tibialis anterior muscle (*m. tibialis anterior*), femoral adductor muscle (*m. adductor femoralis*), ambiens muscle (*m. ambiens*) external flexor of the tibia muscle (*m. flexor tibialis externus*), gastrocnemius muscle (*m. gastrocnemius*), extensor digitorum longus muscle (*m. extensor digitorum longus*), fibularis longus muscle (*m. fibularis longus*), fibularis brevis muscle (*m. fibularis brevis*), flexor digitorum superficialis muscle (*m. flexor digitorum superficialis*), flexor digitorum longus muscle (*m. flexor digitorum longus*), and pronator quadratus muscle (*m. pronator quadratus*). Labels: I—digit I; II—digit II; III—digit III; IV—digit IV; and V—digit V. The figure on the left illustrates the proximal part of the limb, located closer to the body axis. The central illustration depicts the dorsal aspect of the limb, including the dorsal surface of the pes (*dorsum pedis*). The figure on the right presents the limb in lateral view (*pars lateralis*). Images of the pelvic limb are also available in Supplementary Material. Illustration created by A. Tomańska.

In the femoral region, there are several muscles that play a key role in the movements of the limb (Figure 4). The adductor femoralis muscle (*musculus adductor femoralis*) is responsible for the adduction of the limb toward the hip, while the ambiens muscle (*m. ambiens*), consisting of the dorsal and ventral heads, likely affects postural stability and limited abduction of the limb, allowing for knee extension. The ambiens muscle, consisting of two heads, plays a key role in stabilizing and coordinating the movements of the pelvic limb. The dorsal head attaches to the ventral edge of the acetabulum, while the ventral head is positioned anteriorly, both converging to the femur at the intertrochanteric fossa, providing important support to maintain the limb’s posture relative to the body.

The caudofemoralis muscles (*mm. caudofemoralis brevis et longus*) are involved in hip extension and limb abduction by knee flexion. The femorotibialis muscle (*m. femorotibialis*) supports hip flexion and knee extension, while the puboischiofemoralis muscle (*m. puboischiofemoralis*) helps in the precise adjustment and fine positioning of the limb relative to the hip joint [16]. The pubotibialis muscle (*m. pubotibialis*), which has both dorsal and ventral heads, allows for hip flexion and rotation of the limb, playing an important role in balance maintenance. The coordinated action of the various sections of the puboischiofemoralis muscle allows the animal to move efficiently and capture prey. Furthermore, the complex structure of this muscle, along with other lower limb muscles, may play a role in distributing pressure across the limb, potentially contributing to its sensory functions. The puboischiotibialis muscle (*m. puboischiotibialis*) is responsible for hip flexion and enables the external rotation of the limb [16].

In the crural region, which includes the muscles of the lower leg, the following muscles are significant. The extensor digitorum longus muscle (*m. extensor digitorum longus*) is responsible for extending the toes and dorsiflexing the ankle joint, as well as flexing digits. The flexor digitorum longus muscle (*m. flexor digitorum longus*) is involved in the plantarflexion of the ankle joint. The flexor tibialis externus muscle (*m. flexor tibialis externus*) and flexor tibialis internus muscle (*m. flexor tibialis internus*), which have medial and superficial heads, work together in flexing the knee and stabilizing the lower limb. The tibialis anterior muscle (*m. tibialis anterior*) is essential for the dorsiflexion of the pes and controlling its position during movement [16].

The pelvic region includes muscles that stabilize and support the movements of the hip and knee joints. The gastrocnemius muscle (*m. gastrocnemius*), which attaches to the calcaneus, plays an important role in plantarflexing the pes and extending the leg. The iliofibularis muscle (*m. iliofibularis*) stabilizes the pelvic region and the femur during movement, allowing for hip abduction. The iliofibular muscle, originating from the posterior abdominal margin of the femur, forms a broad tendon that attaches to the proximal tibia, while the femorotibial muscle forms two strong tendons on the lateral surface of the femur, with distal attachments on the back of the tibia near the knee patella, aiding in powerful movement and balance. Additionally, the iliotibialis muscle (*m. iliotibialis*) acts as an extensor of the knee joint [16].

In the peroneal region, the fibularis brevis and longus muscles (*mm. fibularis brevis et longus*) are responsible for the plantarflexion of the ankle joint and stabilization of this part of the limb. The fibularis longus plays a key role in the plantarflexion of the tarsus, while the fibularis brevis contributes to both plantarflexion and eversion. These muscles play an important role in precise pes movements, especially when changing directions.

The flexor digitorum superficialis muscle (*m. flexor digitorum superficialis*), the pronator quadratus muscle (*m. pronator quadratus*), located within the ankle joint, is responsible for pronation of the pes. The flexor hallucis longus muscle (*m. flexor hallucis longus*) enables the flexion of the first toe, stabilizing the manipulative movements of the toes and their positioning on the ground [16].

The long extensor muscle of the toes connects to the dorsomedial part of the femoral condyle and the dorsolateral surface of the second and third metatarsal bones. This anatomical feature enables the Komodo dragon to control multiple toes simultaneously, which is particularly useful for hatchlings and juveniles, particularly during their brief arboreal phase, requiring precise manipulation of the pes while climbing or navigating narrow surfaces.

All of these muscles work together to enable complex and precise movements of the limb in various planes (Figure 4) [17].

**Figure 5:**
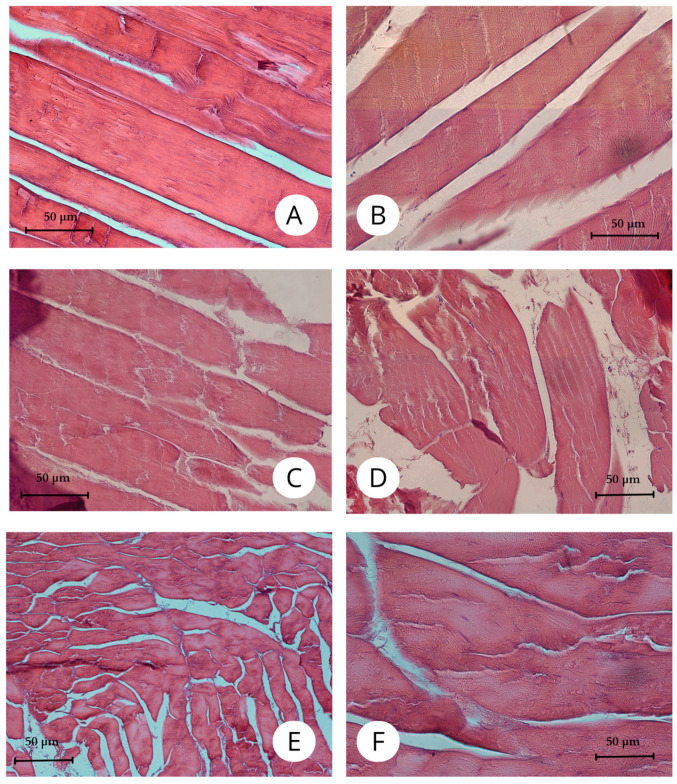
Histological images of selected pelvic limb muscles (HE staining). (**A**)—ambiens muscle (*m. ambiens*, dorsal head), Mag 200×; (**B**)—puboischiofemoral muscle (*m. puboischiofemoralis*), Mag 200×; (**C**)—flexor digitorum longus muscle (*m. flexor digitorum longus*), Mag 200×; (**D**)—superficial flexor of the tibia muscle (*m. flexor tibialis superficialis*), Mag 200×; (**E**)—femorotibial muscle (*m. femorotibialis*), Mag 200×; (**F**)—pubotibial muscle (*m. pubotibialis*), Mag 200×.

**Figure 6:**
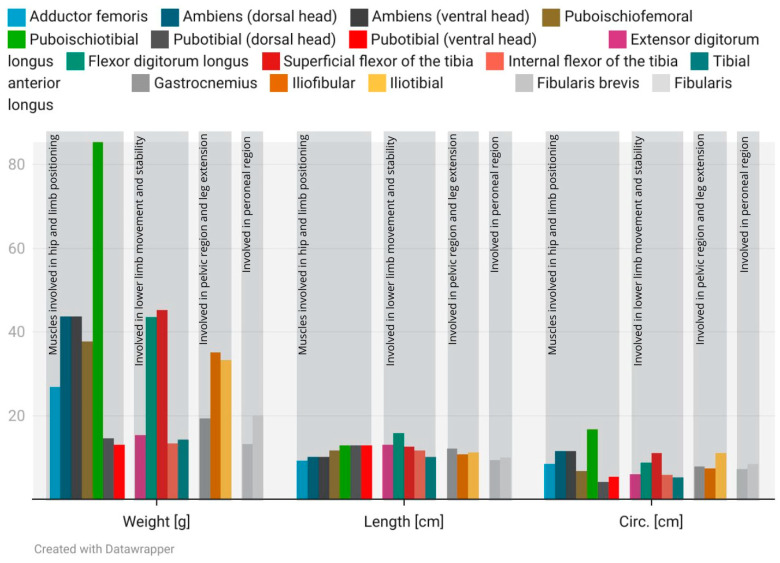
The distribution of muscle fiber diameters (µm, N = 100), circumference (cm), length (cm), and weight (g) across the various muscles of the pelvic limbs is presented. The data were visualized using Datawrapper and correspond to the information detailed in Table 1.

Histological examination revealed a diverse architecture of muscle fibers (Figure 5). The largest fiber diameters were observed in the lower leg flexors, with fiber diameters in the pelvic limbs ranging from approximately 11 to 220 µm (Table 1). The muscles analyzed in this study exhibit varying fiber diameters, suggesting significant structural and functional diversity (Figure 5 and Figure 6).

The flexor digitorum longus muscle shows considerable heterogeneity, with an average fiber diameter of 77.64 µm and variability ranging from 18.18 to 267.78 µm. This suggests that this muscle has a highly variable structure, likely reflecting its diverse functional roles. Similarly, the superficial flexor of the tibia muscle has an average diameter of 79.50 µm, with variability ranging from 24.90 to 220.67 µm, indicating substantial structural variation. The internal flexor of the tibia muscle shows even greater variability, with an average diameter of 88.44 µm and variability ranging from 33.43 to 160.77 µm, further highlighting the diverse structural needs of these muscles.

In contrast, the femorotibial muscle has a more uniform structure, with an average diameter of 32.89 µm and a smaller variability range of 15.32 to 47.15 µm. This suggests that the femorotibial muscle is more specialized in its function. The anterior tibial muscle exhibits moderate variability, with an average diameter of 47.96 µm and variability ranging from 15.15 to 98.48 µm, suggesting a balanced functional and structural diversity. The iliotibial muscle has an average diameter of 47.09 µm, with variability spanning from 16.97 to 90.97 µm, further supporting the idea of moderate variability within this group.

The gastrocnemius muscle shows moderate variability in fiber diameter, with an average of 41.84 µm and variability ranging from 21.14 to 85.73 µm. Similarly, the iliofibularis muscle has an average diameter of 42.78 µm, with variability ranging from 19.81 to 89.18 µm, and the puboischiofemoral muscle shows an average diameter of 60.37 µm, with variability ranging from 11.62 to 159.49 µm. These muscles present a moderate average diameter, with a notable range of variability, suggesting a relatively diverse structural organization.

On the other hand, the fibularis brevis muscle has an average diameter of 36.88 µm, with variability ranging from 14.76 to 87.67 µm, showing a more uniform structure. The fibularis longus muscle also demonstrates a relatively uniform structure, with an average diameter of 35.91 µm and variability ranging from 14.87 to 62.83 µm. Both of these muscles exhibit smaller ranges of variability, indicating a more consistent fiber structure.

The puboischiotibial muscle has an average diameter of 89.12 µm, with variability ranging from 76.79 to 101.45 µm, suggesting a more uniform structure with less variability. The pubotibial muscle has an average fiber diameter of 48.98 µm, with variability ranging from 41.77 to 56.19 µm, reflecting relatively low variability. Additionally, the pubotibial muscle (ventral head) shows an average diameter of 78.32 µm, with variability ranging from 68.98 to 86.66 µm, indicating a more consistent structure.

The pelvic limb musculature of *Varanus komodoensis* exhibits variation in muscle mass relative to body weight and in the ratios of the muscle fiber diameter to muscle length. Several muscles, including the pubotibial muscle (ventral head), anterior tibial, fibularis brevis, fibularis longus, gastrocnemius, internal flexor of the tibia, extensor digitorum longus, flexor digitorum longus, ambiens (dorsal head), iliofibular, iliotibial, and adductor femoral, account for modest proportions of the total muscle mass, typically ranging between 0.04% and 0.07% of the body weight.

In contrast, the puboischiotibial muscle stands out with the highest mass-to-body weight ratio, constituting 0.28%, while muscles such as the flexor digitorum longus, superficial flexor of the tibia, and pubotibial (dorsal head) exhibit intermediate contributions, ranging from 0.09% to 0.15%. When examining muscle fiber diameter relative to muscle length, notable patterns emerge. The internal flexor of the tibia demonstrates the highest ratio (7.56), followed by the pubotibial (ventral head) (6.07), superficial flexor of the tibia (6.31), and puboischiotibial muscle (6.91).

**Figure 7:**
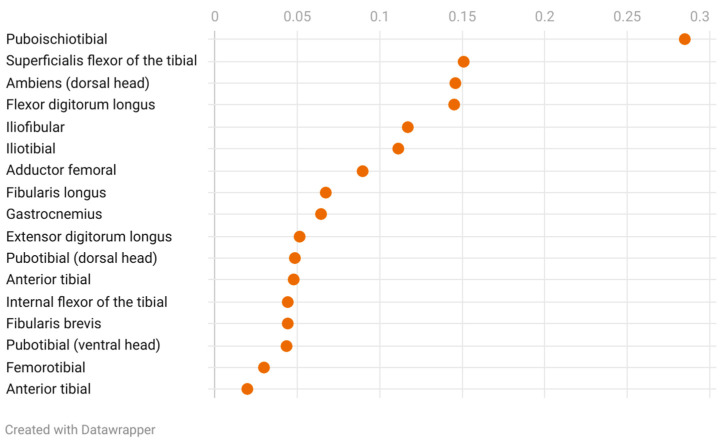
Muscle weight-to-body weight ratios for the pelvic limb musculature of *Varanus komodoensis* specimen: adductor femoral, 0.089; ambiens (dorsal head), 0.146; iliofibular, 0.117; iliotibial, 0.111; femorotibial, 0.029; extensor digitorum longus, 0.051; anterior tibial, 0.019; flexor digitorum longus, 0.145; superficialis flexor of the tibial, 0.151; internal flexor of the tibial, 0.044; gastrocnemius, 0.064; fibularis brevis, 0.044; fibularis longus, 0.067; anterior ibial, 0.047; puboischiotibial, 0.284; pubotibial (dorsal head), 0.048; and pubotibial (ventral head), 0.044. Visualized using Datawrapper.

**Figure 8:**
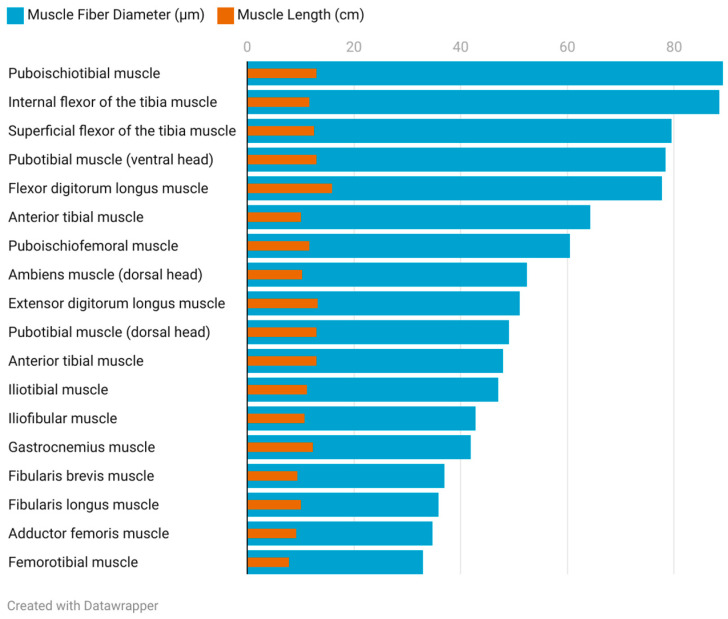
Muscle fiber diameter-to-muscle length ratios in the pelvic limb musculature of the examined *Varanus komodoensis* specimen: adductor femoris, 3.77; ambiens (dorsal head), 5.14; extensor digitorum longus, 3.90; femorotibial, 4.22; flexor digitorum longus, 4.91; anterior tibial, 3.72; superficial flexor of the tibia, 6.31; internal flexor of the tibia, 7.56; gastrocnemius, 3.43; iliofibular, 3.96; iliotibial, 4.20; fibularis brevis, 3.92; fibularis longus, 3.58; puboischiofemoral, 5.16; puboischiotibial, 6.91; pubotibial (dorsal head), 3.80; pubotibial (ventral head), 6.07; and anterior tibial, 6.36. Visualized using Datawrapper.

Conversely, muscles such as the gastrocnemius (3.43), anterior tibial (3.72), and fibularis longus (3.58) exhibit lower fiber-to-length ratios, along with the adductor femoral (3.77) and extensor digitorum longus (3.90) (Figure 6, Figure 7 and Figure 8).

The data presented refer to a single specimen of the Komodo dragon, so they should not be generalized to the entire species. A description of normal anatomy should be based on broader studies and further research.

## 4. Discussion

In terrestrial locomotion, the pelvic limb serves as the main propulsive force, particularly in reptiles [22]. Variations in limb morphology play a crucial role in determining both force generation and speed. Species with longer limbs tend to have longer strides and a lower stepping frequency, which leads to increased vertical forces during the stance phase, simultaneously reducing the energy required for movement [23].

The anatomical data we collected provide foundational insights into the musculoskeletal structure of this species. Muscles of the pelvic limb could be crucial in managing ground reaction forces, supporting a sprawling limb posture, and aligning the femur, lower leg, and pelvis. Although we did not directly analyze tail movement in this study, previous research by our team suggests that these flexors might contribute to tail coordination [24].

Our findings are in line with observations by Dick and Clemente, who reported that in the pelvic limb, nine out of twenty-one muscles are primarily active during the stance phase, five during the swing phase, and seven are active in both phases. This pattern reveals that about 25% more muscles are involved in stance-related activities, highlighting their importance in providing support and stability during movement [17].

The gathered data may suggest that the Komodo dragon’s morphology is adapted to its environment, with rapid running potentially playing a minor role in its behavior, as the species primarily relies on other modes of locomotion. This observation indicates that the species’ behavior is better suited to waiting and ambushing prey rather than engaging in prolonged pursuit. Considering these points, one can argue that the hindlimbs provide a strong driving force for movement, yet the primary navigation of movement is controlled by the forelimbs and tail. The force generated by the muscles of the hindlimbs allows the animal to traverse varied terrain effectively. The Komodo dragon, despite being a prominent terrestrial predator, does not exhibit specific adaptations for speed. In reptiles, running is not solely dependent on the limbs; it also involves the muscles of the back, as well as the flexibility of the spine and pelvis. Some lizards are capable of sprinting at speeds comparable to mammals. However, for certain species of lizards, high-speed locomotion necessitates elevating the body on the hindlimbs [25]. They utilize lateral body bending during running, a key factor in the ongoing scientific debate about the role of limbs in determining the mechanics of locomotion across different animals [26]. For instance, in mammals, it is well known that species adapted for high-speed locomotion, such as cheetahs and racing greyhounds, demonstrate limb elongation and reduced muscle mass in the pelvic region. This trend is evident in the scaling of the pelvic limb, where increased stride length plays a crucial role in efficient movement, as seen in these fast-moving species [27]. The observed anatomical features in the Komodo dragon may align with theories proposed for large mammals, where reduced locomotor efficiency helps maintain consistent biomechanical stresses, facilitating adaptive bone remodeling [28]. This suggests a distinct strategy for locomotion development in the Komodo dragon, which warrants further investigation. Future studies, particularly the publication of micro-anatomical data and comparative analysis of the skeletal system, are essential to clarify these adaptations [29].

The musculature of the Komodo dragon exhibits notable differences between the forelimbs and hindlimbs, particularly in muscle mass and functional variability [21]. The hindlimbs are dominated by larger muscles, with greater variability in fiber width among muscles with larger diameters. This variability highlights the adaptability of the hindlimb musculature, enabling the animal to perform specialized movements crucial for navigating diverse terrains and reducing the risk of injury on uneven surfaces or inclines. The greatest structural variability occurs in muscles involved in complex movements or those requiring adaptation to diverse biomechanical tasks. Muscles with a higher ratio of fiber diameter to muscle length, such as the internal flexor of the tibia (*M. flexor tibialis internus*) or the puboischiotibial muscle (*M. puboischiotibialis*), demonstrate the ability to generate greater force over shorter ranges of motion. This characteristic is especially vital for the Komodo dragon when traversing uneven terrain. Conversely, lower ratios in muscles like the gastrocnemius (*M. gastrocnemius*) or fibularis longus (*M. fibularis longus*) suggest more balanced functions, such as stabilizing the limb during walking or running. Additionally, greater variability in fiber diameters is correlated with a broader range of potential muscle functions, reflecting their diverse roles in locomotion and stability [30].

The long caudofemoral muscle plays a key role in understanding the locomotion physiology of this animal and therefore requires a distinct methodological approach. In this context, it is worth referencing a study conducted by White R. on *Alligator mississippiensis*. This approach should include the dissection of the tail and pelvic limb area, as well as a detailed anatomical description of the pelvis, preceded by a computed tomography scan. Additionally, it will be necessary to collect precise osteological data from this area, considering potential sex-based differences in anatomical features. White’s studies have shown that in crocodilians, this muscle functions as the primary hip joint extensor and pelvic limb retractor, with its action supported by the *m. transversus perinei*. Tracing the tendons of the *m. caudofemoralis longus* in *A. mississippiensis* revealed its connection to femur rotation but also highlighted other possible functions. Together with the *M. gastrocnemius*, it can contribute to knee flexion, while on its own, it may influence pelvic limb retraction by drawing it closer to the body’s midline. During contraction, the force generated by this muscle is transmitted to the femur, tarsal bones, and metatarsals, influencing the movement of the entire limb. In *A. mississippiensis*, positioning the limb parallel to the body’s axis, combined with the supporting role of the tail’s lateral movements, may enhance the efficiency of tail contractions, playing a crucial role in the animal’s swimming behavior [31].

When examining the ratio of muscle fiber diameter to muscle length, most muscles show a consistent average of 0.428 (SD = 0.102), with a median of 0.393. However, muscles such as the long extensor of the toes (*M. extensor digitorum longus*) (0.491), ambiens muscle (*M. ambiens*, dorsal head) (0.513), and internal flexor of the tibia (*M. flexor tibialis internus*) (0.756) deviate significantly, the latter displaying a value nearly double the median. This suggests specialized roles for these muscles, reflecting their adaptation to different biomechanical demands.

The least developed muscles, such as the anterior tibial muscle (*M. tibialis anterior*) (<0.02%) and femorotibial muscle (*M. femorotibialis*) (<0.03%), contrast sharply with the most developed muscle, the puboischiotibial muscle (*M. puboischiotibialis*) (0.284%), marking a nearly fifteenfold difference. This disparity underscores the importance of specific muscle groups in supporting the Komodo dragon’s ecological role and movement strategies. The muscle mass relative to body weight shows significant diversity, which is indicative of the Komodo dragon’s foraging strategy and its use of its individual range [32].

Jessop et al. demonstrated that Komodo dragons differ from other apex predators. Their reliance on ambush hunting may affect prey population regulation, as shown by the lack of a significant impact on prey population growth rates despite the dragons’ higher biomass and energy consumption [33]. On islands with fewer prey, the dragons tend to have smaller body sizes, indicating developmental plasticity. Additionally, Komodo dragons in captivity exhibit better cognitive abilities and more complex behavioral repertoires compared to other scaly lizards. Throughout their development, from hatchlings to adults, they target different types of prey [34].

The Komodo dragon’s morphology prioritizes strength and precision over speed. The relatively modest development of certain muscles is combined with a functional focus on larger, adaptable hindlimb muscles. This indicates that rapid running is not a primary trait of the species. Instead, it likely relies on ambush predation, with less emphasis on sustained pursuit. While the hindlimbs provide substantial force for movement, locomotion is mainly controlled by the coordination of the forelimbs and tail, supporting stability and power across challenging terrains. The ratio of the length of the proximal segments (hip, thigh, and shank) to the length of the foot (1.98) indicates a balance between the force-generating capacity of the hindlimbs and the functional role of the foot in locomotion, further reflecting the dragon’s specialized movement mechanics.

Further research is necessary to fully understand the species’ adaptations. Nonetheless, our observations can provide a foundation for discussions on the species’ typical anatomy, as well as other relevant aspects. These findings may also shed light on the Komodo dragon’s evolutionary traits, emphasizing strength, versatility, and hunting effectiveness rather than speed, in line with its ecological role and predatory behavior.

## 5. Conclusions

The musculoskeletal system of *Varanus komodoensis* is uniquely adapted for endurance, strength, and stability, rather than speed. The overall anatomy of the pelvic limb, which includes a range of muscles such as the pubotibial, femoral adductor, gastrocnemius, and tibialis anterior muscles, is designed to provide the necessary force and support for dynamic movements in various environmental contexts. These anatomical features, including the robust musculature and complex joint structure, allow the species to excel in activities requiring power, stability, and control, such as climbing, running, or bracing.

Histologically, the muscle fibers exhibit notable variability in their diameters, reflecting pronounced structural heterogeneity. The largest fiber diameters were found in the flexors of the lower leg, particularly the flexor digitorum longus muscle, which shows considerable variation. This diversity suggests that these muscles are capable of performing a wide range of movements, from precise, quick actions to powerful, sustained contractions. Such variability supports the idea that the muscles of *Varanus komodoensis* are adapted to handle fluctuating forces during locomotion, with some muscles specialized for explosive movements, while others provide steady, long-term support.

In particular, the flexor digitorum longus muscle, with its wide range of fiber diameters (18.18 to 267.78 µm), is well suited for dynamic, high-force movements such as rapid changes in direction or securing grip, which are essential for climbing and hunting. Similarly, the tibial flexors muscles (superficial and internal) show considerable variability in their fiber structure, enabling them to adjust force output for both fine motor control and powerful pushing motions. These muscles are crucial for stabilizing the lower limb during movement and maintaining posture under varying loads.

On the other hand, muscles such as the femorotibial, pubotibial, and puboischiotibial exhibit more uniform fiber structures, suggesting a specialization for endurance and sustained force. These muscles are likely responsible for tasks requiring consistent, repetitive actions, such as maintaining stability during walking or running, where long-term exertion and posture control are critical. The presence of sesamoid bones in the knee joint and other anatomical features like thick, compact musculature in the femoral region support the idea that the musculoskeletal system is structured to optimize the species’ strength and stability rather than speed. These features contribute to the animal’s ability to perform heavy, load-bearing movements and enhance the durability of the joints under repeated stress, which is crucial for a species that relies on both mobility and endurance to navigate its environment and hunt prey.

Our findings align with the anatomical descriptions provided by Dick and Clemente [17], confirming the detailed structure of the muscles and joints that facilitate stability and strength. Muscles like the adductor femoralis, ambiens, and caudofemoralis, for example, play important roles in both limb positioning and the stabilization of the body during locomotion. This complex interaction between the musculature and skeletal structure ensures that the animal can efficiently perform high-stress activities like climbing, hunting, and bracing while maintaining balance and avoiding injury.

The use of a single specimen of the Komodo dragon, while providing detailed anatomical insights, limits the representativeness of the results. Studies based on a single individual may not capture the full intraspecific variation within the population, such as differences in sex, age, health, or geographical origin. Given the unique nature of the research material, future studies should involve a larger sample of individuals and encourage the sharing of morphological data among researchers, allowing for a more comprehensive understanding of the species’ anatomy and function. This is particularly important when studying locomotor mechanics. Since our findings are based on one individual, further research should focus on a more detailed analysis of muscle fiber types, which could reveal traits related to strength, speed, and endurance. Attention should also be given to muscle insertion angles and the detailed architecture of each muscle, including its physiological cross-sectional area. Variability in these aspects could highlight adaptations for different types of movement.

The *m. caudofemoralis longus* was not specifically addressed in this study, but it warrants further investigation due to its role in linking the anatomy and function of the pelvis, hindlimb, and tail. Similar anatomical studies have been conducted in other species, such as *Alligator mississippiensis*, with White’s work providing a relevant reference in this context.

In conclusion, the muscular and skeletal systems of *Varanus komodoensis* are highly specialized for strength, stability, and endurance, rather than for speed or agility. The variability in muscle fiber diameters reflects the diverse mechanical demands placed on different muscle groups, with some muscles built for fine control and rapid movements and others adapted for sustained, powerful force. This intricate anatomical and histological structure is essential for the species’ survival in diverse environments and highlights the importance of muscle fiber adaptability in performing complex locomotor tasks. The overall design emphasizes strength, endurance, and stability as the key functional priorities for *Varanus komodoensis’s* movement and survival strategies.

## Figures and Tables

**Table 1:** The measurements of selected muscles including the weight (g), length (cm), and circumference at the widest part of the muscle belly (circ.) [cm], along with an analysis of muscle fiber diameter (µm), encompassing the mean, minimum, and maximum values (Min/Max), as well as the standard deviation (St. Dev.). For each measurement, a series of 100 repetitions (N = 100) was conducted.

Muscle Measurements	Weight [g]	Length [cm]	Circ. [cm]	Muscle Fiber Diameter (µm, N = 100)		
				Mean	St. Dev.	Min/Max
Adductor femoris muscle	26,818	9.2	8.4	34.63	4.52	19.17/90/89
Ambiens muscle (dorsal head)	43,739	10.2	11.5	52.44	7.35	24.68/92.04
Extensor digitorum longus muscle	15,322	13.1	6.0	51.11	8.11	22.67/89.28
Femorotibial muscle	8.803	7.8	6.1	32.89	7.32	15.32/47.15
Flexor digitorum longus muscle	43,525	15.8	8.8	77.64	22.78	18.18/267.78
Anterior tibial	5.762	12.9	3.5	47.96	7.28	15.15/98.48
Superficial flexor of the tibia muscle	45,290	12.6	11.1	79.50	20.96	24.90/220.67
Internal flexor of the tibia muscle	13,295	11.7	5.8	88.44	13.47	33.43/160.77
Gastrocnemius muscle	19,332	12.2	7.8	41.84	6.85	21.14/85.73
Iliofibular muscle	35,059	10.8	7.4	42.78	6.62	19.81/89.18
Iliotibial muscle	33,214	11.2	11.0	47.09	7.52	16.97/90.97
Fibularis brevis muscle	13,171	9.4	7.3	36.88	6.27	14.76/87.67
Fibularis longus muscle	20,145	10.03	8.4	35.91	10.42	14.87/62.83
Puboischiofemoral muscle	37,765	11.7	6.8	60.37	14.87	11.62/159.49
Puboischiotibial muscle	85,334	12.9	16.8	89.12	12.33	76.79/101.45
Pubotibial muscle	14,538	12.9	4.2	48.98	7.21	41.77/56.19
Pubotibial muscle (ventral head)	13,072	12.9	5.4	78.32	8.34	68.98/86.66
Anterior tibial muscle	14,230	10.1	5.2	64.23	12.87	51.36/77.10

## Data Availability

There are no other details regarding where data supporting the reported results can be found, including links to publicly archived datasets analyzed or generated during this study.

## Supplementary Materials

The following supporting information can be downloaded at https://www.mdpi.com/article/10.3390/ani15010035/s1: Photographs of the pelvic limb of the Komodo dragon (*Varanus komodoensis*) taken during dissection. S.1—dorsal view; S.2—lateral view; S.3—medial view. Images were captured using a Nikon D90 camera with an aperture setting of f/8.
