# Supplementary material for: The Musculoskeletal Anatomy of the Komodo Dragon’s Hindlimb (Varanus komodoensis, Varanidae)
Source: Animals (Basel). 2024 Dec 26;15(1):35. doi: 10.3390/ani15010035 (PMC11718905; doi:10.3390/ani15010035)
Supplement: Supplementary file 1 [file animals-15-00035-s001.zip › animals-3365598-supplementary.pdf]

Supplementary Materials

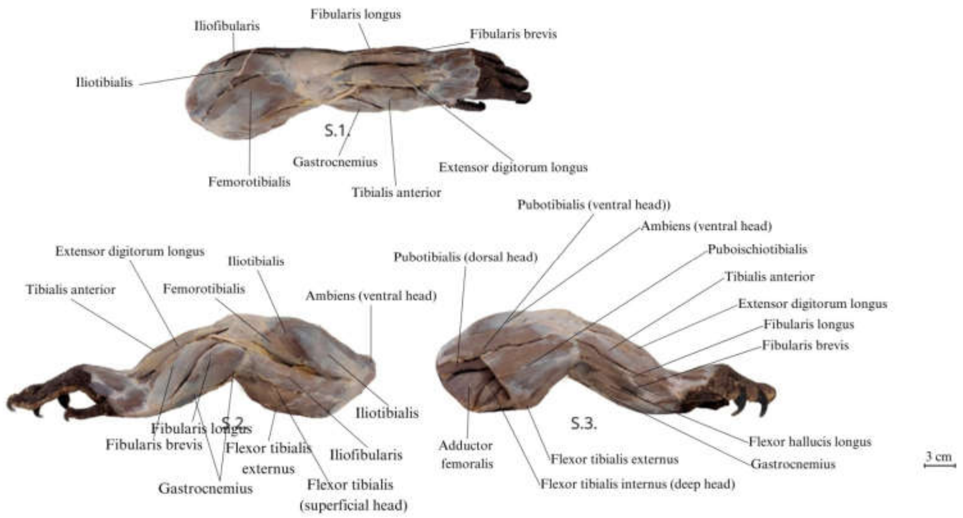

Photographs of the pelvic limb of the Komodo dragon (*Varanus komodoensis*) taken during dissection. S.1 – Dorsal view, S.2 – Lateral view, S.3 – Medial view. Images were captured using a Nikon D90 camera with an aperture setting of f/8.
